# Biomarkers of intake for coffee, tea, and sweetened beverages

**DOI:** 10.1186/s12263-018-0607-5

**Published:** 2018-07-04

**Authors:** Joseph A. Rothwell, Francisco Madrid-Gambin, Mar Garcia-Aloy, Cristina Andres-Lacueva, Caomhan Logue, Alison M. Gallagher, Carina Mack, Sabine E. Kulling, Qian Gao, Giulia Praticò, Lars O. Dragsted, Augustin Scalbert

**Affiliations:** 1 International Agency for Research on Cancer (IARC), Nutrition and Metabolism Section, Biomarkers Group, 150 Cours Albert Thomas, F-69372 Lyon CEDEX 08, France; 2 Biomarkers and Nutrimetabolomics Laboratory, Department of Nutrition, Food Sciences and Gastronomy, Campus Torribera, Faculty of Pharmacy and Food Sciences, University of Barcelona, Barcelona, Spain; 3 CIBER de Fragilidad y Envejecimiento Saludable (CIBERFES), Instituto de Salud Carlos III, Barcelona, Spain; 4 Nutrition Innovation Centre for Food and Health (NICHE), Biomedical Sciences Research Institute, Ulster University, Cromore Road, Coleraine, Northern Ireland; 5 Department of Safety and Quality of Fruit and Vegetables, Federal Research Institute of Nutrition and Food, Max Rubner-Institut, Karlsruhe, Germany; 6 Department of Nutrition, Exercise and Sports, Faculty of Science, University of Copenhagen, Copenhagen, Denmark

**Keywords:** Non-alcoholic beverages, Coffee, Tea, Sugar-sweetened beverages, Low-calorie-sweetened beverages, Biomarkers, Intake

## Abstract

Non-alcoholic beverages are important sources of nutrients and bioactive compounds that may influence human health and increase or decrease the risk of chronic diseases. A wide variety of beverage constituents are absorbed in the gut, found in the systemic circulation and excreted in urine. They may be used as compliance markers in intervention studies or as biomarkers of intake to improve measurements of beverage consumption in cohort studies and reveal new associations with disease outcomes that may have been overlooked when using dietary questionnaires. Here, biomarkers of intake of some major non-alcoholic beverages—coffee, tea, sugar-sweetened beverages, and low-calorie-sweetened beverages—are reviewed. Results from dietary intervention studies and observational studies are reviewed and analyzed, and respective strengths and weaknesses of the various identified biomarkers discussed. A variety of compounds derived from phenolic acids, alkaloids, and terpenes were shown to be associated with coffee intake and trigonelline and cyclo(isoleucylprolyl) showed a particularly high specificity for coffee intake. Epigallocatechin and 4′-*O*-methylepigallocatechin appear to be the most sensitive and specific biomarkers for green or black tea, while 4-*O*-methylgallic acid may be used to assess black tea consumption. Intake of sugar-sweetened beverages has been assessed through the measurement of carbon-13 enrichment of whole blood or of blood alanine in North America where sugar from sugarcane or corn is used as a main ingredient. The most useful biomarkers for low-calorie-sweetened beverages are the low-calorie sweeteners themselves. Further studies are needed to validate these biomarkers in larger and independent populations and to further evaluate their specificity, reproducibility over time, and fields of application.

**Electronic supplementary material:**

The online version of this article (10.1186/s12263-018-0607-5) contains supplementary material, which is available to authorized users.

## Background

Human adults consume about 3 to 4 l of water per day, which originate from plain water, solid foods, and other beverages like coffee, tea, soda, fruit juices, sport and energy drinks, milk, and alcoholic beverages [1]. Beside water, non-alcoholic beverages such as coffee and tea are also a source of other constituents like caffeine, chlorogenic acids, diterpenes, and diketopiperazines in coffee [2], theanine, catechins, theaflavins, thearubigins and flavonols in tea [3], sucrose or high-fructose corn syrup (HFCS) in sodas, and low-calorie sweeteners (LCSs) in low-calorie-sweetened beverages (LCSBs) which may influence health in a positive or negative way.

Although coffee drinking was initially thought to induce negative effects on health, for example, increasing blood pressure and the risk of cardiovascular disease risk in some cohorts [4, 5], the main interest in recent years is the potential for positive health effects. These include plausible reductions in risk of type 2 diabetes, Parkinson disease, Alzheimer’s disease, and liver and colorectal cancer [6–10]. Tea consumption has been associated with a reduction of the risk of chronic diseases and more particularly cardiovascular diseases, type 2 diabetes, cancers, and neurodegenerative diseases [11–14]. Compounds like sugars in sodas may provide an excess of energy and increase the risk of obesity and associated diseases [15]. Given the overconsumption of free sugars, particularly from SSBs, the use of LCSBs has increased over the years as a strategy for reducing the free sugar content of the diet while maintaining palatability and research interest to date has focused on assessing the effect of LCSB consumption on a variety of health outcomes such as metabolic health, weight management, and renal impairment [16, 17].

Despite large efforts in unraveling these health effects of non-alcoholic beverages on health in epidemiological studies, some of these effects are not fully consistent between studies, and it has been suggested that this lack of agreement could be associated with the traditional methods used to assess beverage consumption [18]. Beverage consumption is most often measured with self-administered questionnaires, and these measurements are known to be prone to random or systematic errors which may prevent the detection of associations of intake with disease outcomes [19]. These errors are linked to limitations in the capacity of subjects to accurately describe their beverage intake and to difficulties in accounting for variations in the nature of the beverages consumed within or between populations. For example, concentrations of polyphenols in coffee and in tea vary with the type of coffee beans or tea leaves and with the method of coffee brewing or tea leaf infusion, information most often not recorded in dietary questionnaires.

In contrast to questionnaires, biomarkers are objective measures that provide estimates of beverage intake [20–22]. They are directly derived from beverage constituents absorbed in the gut during digestion. Thus, they provide more direct estimates of exposure to beverage constituents and are notably independent of the dilution of the beverages, or on any aspect of the recipes used for their preparation. Biomarkers of beverage intake can be identified using two separate approaches. Initially, biomarkers were hypothesized based on knowledge of beverage constituents and their metabolism. Beverages are fed to volunteers in controlled intervention studies and known metabolites of interest measured in biofluids to test for increases with intake. Until a few years ago, this targeted approach was the only viable method of biomarker discovery. More recently, metabolome profiling has taken precedence as a more comprehensive and agnostic approach. Rather than measuring known compounds only, biofluids are profiled to measure the relative intensities of as many signals as can be detected, either by nuclear magnetic resonance (NMR) or mass spectrometry coupled to liquid or gas chromatography (LC-MS or GC-MS). Signals associated with intake of the target food across the most possible subjects are retained as candidate biomarkers.

Initial metabolome profiling studies used an intervention design with a standardized dose and controlled diet, but more sensitive analytical techniques have enabled the analysis of biofluids from free-living subjects who have reported their beverage intakes via dietary questionnaires. This approach has some advantages and limitations. It encourages the discovery of biomarkers that are valid in the presence of interfering dietary factors and that also account for differences in the time period between beverage intake and biofluid collection. On the other hand, finding new biomarkers may be prevented by the limited accuracy of self-reported dietary intake data. Whatever the discovery approach, biomarkers can only be considered valid for the populations and biofluids in which they are discovered as different populations drink different brands or brews of a beverage with different compositions.

The purpose of this review is to describe the various biomarkers proposed to evaluate intake of some non-alcoholic beverages, including coffee, tea, sugar-sweetened beverages (SSBs), and LCSBs, all beverages that have raised much interest for being widely consumed worldwide and for their effects on the risk of various chronic diseases. Other non-alcoholic beverages such as fruit juices and milk are discussed in other chapters in this special volume. For each beverage, the main metabolites formed from their constituents and identified in blood or urine in human dietary intervention studies as putative biomarkers are first described. Their eventual detection in observational studies and respective value as intake biomarkers is then discussed.

## Materials and methods

The reviewing process made was described in details recently [23] and use all elements of the PRISMA statement [24] that were relevant for a literature search on dietary biomarkers. Original research papers and reviews were searched for in PubMed, Scopus, and ISI Web of Knowledge using the following search terms: (biomarker* OR marker* OR metabolite* OR biokinetics OR biotransformation) AND (human* OR men OR women OR male OR female OR patient* OR volunteer* OR participant*) AND (urine OR plasma OR serum OR blood OR excretion) AND (intake OR meal OR diet OR ingestion OR consumption OR eating OR drink* OR administration) AND (‘name of beverage’). Name of beverages were (coffee), (tea), or (diet soda OR diet beverage* OR soft drink* OR artificial* sweet* beverage* OR low sugar beverage* OR low calorie sweet* beverage*).

The research was limited to papers in English language, and no restrictions were applied to publication date. End-date of search was April 2016, January 2017, June 2016, and December 2016 for coffee, tea, SSBs, and LCSBs, respectively. The research papers identifying or using potential biomarkers of intake for the foods were selected by one or more skilled researchers from the list of retrieved references in the process outlined in Additional file 1: Figure S1. Additional papers were identified from reference lists in these papers and from reviews or book chapters identified through the search. For each potential biomarker identified, an additional search was conducted with (“the name and synonyms of the compound” OR “the name and synonyms of any parent compound”) AND (biomarker* OR marker* OR metabolite* OR biokinetics OR biotransformation) to identify potential other foods containing the biomarker or its precursor. In this second step, Scifinder and Google Scholar were also used as search platforms, as well as the databases listed above. This second search was used to evaluate the apparent specificity of the marker.

## Results and discussion

Biomarkers have been systematically searched for in the scientific literature independently for the four types of non-alcoholic beverages (coffee, tea, SSBs, and LCSBs). The literature was particularly abundant for biomarkers of coffee and tea intake (Additional file 1: Figure S1). Results are successively presented below.

### Coffee biomarkers

#### Coffee metabolites in controlled intervention studies

Many controlled intervention studies have been performed on coffee to study the metabolism of known coffee constituents (Table 1). Most aimed to study the metabolism of caffeoylquinic acid derivatives. Typically, in these studies, a small number of subjects consumed coffee after a washout period, and blood or urine samples were taken at intervals for analysis. In early studies, metabolites were quantified by HPLC after enzymatic hydrolysis of glucuronide and sulfate conjugates. Isoferulic acid concentrations most markedly increased in urine samples taken periodically of volunteers repeatedly dosed with coffee and was thus proposed as a potential intake biomarker [25]. Another such intervention indicated that caffeic acid, as well as the microbial metabolite *m*-coumaric acid, appears in 24-h urine after coffee intake [26]. With mass spectrometers, many more metabolites derived from coffee chlorogenic acids were later characterized without enzyme treatment. For example, individual caffeic and ferulic acid conjugates were measured by LC-MS in the plasma and urine of subjects fed instant coffee [27]. Dihydroferulic acid 4-*O*-sulfate and dihydrocaffeic acid 3-*O*-sulfate attained the highest plasma concentrations after coffee intake. Dihydrocaffeic acid 3-*O*-sulfate and feruloylglycine were reported as the most sensitive urinary biomarkers of intake. Further, in a double-blind randomized controlled trial investigating bioavailability of chlorogenic acids from coffee, all chlorogenic acid metabolites increased in a dose-dependent manner in plasma and urine after the administration of coffee containing three different levels of chlorogenic acids [28]. Concentrations of caffeic and ferulic acid sulfates were most markedly increased, but coffee intake also caused increases of intact caffeoylquinic acids and sulfated caffeoylquinic acid lactones. 3-Feruloylquinic acid, in both 24-h urine and plasma, was highly correlated with the caffeoylquinic acids consumed from coffee (Spearman *r* = 0.81 and *r* = 0.73, respectively).

**Table 1:** Metabolites identified in human intervention studies on coffee

Beverage	No. subjects	Sample type	Analytical method	Enzymatic hydrolysis	Discriminating metabolites/candidate biomarkers	Reference
Instant coffee (repeated intake)	5	Urine	HPLC	Yes	Ferulic, isoferulic, dihydroferulic, vanillic acids, 3-hydroxyhippuric acid	[25]
Instant coffee	9	Urine	LC-MS	Yes	Chlorogenic acid, caffeic acid, m-coumaric acid	[26]
Coffee	13	Urine	CE-MS	No	Coumaric acid, caffeic acid	[127]
Instant coffee	11	Plasma, urine	LC-MS	No	Ferulic and dihydroferulic acid sulfates (blood and urine), feruloylglycine (urine only)	[27]
Filter coffee	9	Urine	LC-MS	No	Trigonelline, N-methylpyridium	[35]
Filter coffee	13	Plasma	LC-MS	No	Trigonelline, dimethylxanthines, methylxanthines, dihydroferulic acid, dihydrocaffeic acid sulfate, ferulic acid glucuronide, ferulic acid sulfate, ferulic acid, dihydroferulic acid sulfate, dihydroferulic acid glucuronide, N- feruloylglycine	[29]
Instant coffee	11	Plasma, urine	LC-MS	No	3-, 4- and 5-Feruloylquinic acid, 3 and 4-Caffeoylquinic acid lactone sulfate (urine and plasma), isoferulic acid-3-glucuronide (urine only)	[28]
Coffee (repeated intake)	8	Urine	1H-NMR	No	2-Furoylglycine	[30]

Despite providing a wealth of information on potential markers of coffee intake, the main purpose of these controlled intervention studies was usually to investigate the metabolism of hydroxycinnamic acid derivatives rather than search for novel biomarkers of intake. Other authors have followed the appearance of a variety of coffee compounds in blood or urine in subjects administered a standardized dose of coffee [29]. Dihydroferulic acid conjugates, trigonelline, caffeine, and its primary metabolites were found to persist in plasma for long enough that they should never be fully cleared in individuals drinking three cups of coffee over a day. *N*-2-furoylglycine was identified as a promising biomarker of coffee intake after the untargeted NMR profiling of spot urine samples from five volunteers administered a dose of espresso coffee [30]. Highest concentrations were observed two hours after intake. *N*-2-furoylglycine is derived from furans formed during coffee roasting.

#### Biomarkers of coffee intake in observational studies

Markers uncovered in controlled intervention studies may be sensitive but not sufficiently specific to the food of interest since other possible food sources of these metabolites are excluded throughout the intervention. Caffeoylquinic and feruloylquinic acids and their derivatives, for example, are also present in fruits, vegetables, and grains [31]. Although coffee is the principal dietary source, high intake of other confounding foods could lead to inaccurate estimates of intake. Biomarkers lacking specificity are better excluded in observational studies, in which subjects consume their usual diets. Food intake is estimated with dietary questionnaires before blood or urine collection. The first such study on coffee hypothesized that urinary isoferulic acid, a metabolite of caffeoylquinic and caffeic acids, would reflect habitual coffee intake (Table 2) [32]. Study participants, consuming their usual diets, recorded coffee intake via questionnaires, and 24-h pooled urine was collected. Urinary isoferulic acid excretion varied substantially between coffee consumers and was not strongly related to coffee intake as reported by food frequency questionnaire (FFQ) (*r* = 0.26) or 24-h dietary recall (*r* = 0.18). Another study on 53 free-living French subjects showed a stronger correlation (*r* = 0.63, *p* < 0.001) between 5-caffeoylquinic acid concentrations in spot urine and coffee intake, but this association was not statistically significant when tested in 24-h urine [33]. High correlations were observed between coffee intake and caffeic acid (*r* = 0.65), protocatechuic acid (*r* = 0.60), and ferulic acid (*r* = 0.58) concentrations measured in 24-h urine collected in 475 adult participants from the European Prospective Investigation into Cancer and Nutrition (EPIC) cross-sectional study [34].

**Table 2:** Biomarkers of coffee intake discovered in observational studies

Beverage	No. subjects	Sample type	Analytical method	Enzymatic hydrolysis	Discriminating metabolites/candidate biomarkers (HMDB ID if available)	Association with	Reference
Coffee	111	Urine (24 h)	GC-MS	Yes	Isoferulic acid (HMDB0000955)	FFQ	[32]
Coffee	344	Urine (24 h)	GC-MS	Yes	Isoferulic acid (HMDB0000955)	24-HDR	[32]
Coffee	53	Urine (24 h and spot)	LC-MS	Yes	Chlorogenic acid (HMDB0003164), caffeic acid (HMDB0001964)	2-Day dietary record	[33]
Coffee	68	Urine (24 h)	FIA-MS	Yes	Dihydrocaffeic acid (not in HMDB), dihydrocaffeic acid 3-glucuronide (HMDB0041720)	FFQ	[36]
Coffee	39	Urine (morning spot)	LC-MS	No	Atractyligenin glucuronide (not in HMDB), cyclo(isoleucylprolyl) (not in HMDB), trigonelline (HMDB0000875), paraxanthine (HMDB0001860), theobromine (HMDB0002825), theophylline (HMDB0001889), 1-methylxanthine (HMDB0010738), hippuric acid (HMDB0000714), trimethyluric acid (HMDB0002123), 3-hydroxyhippuric acid (HMDB0006116), 5-acetylamino-6-formylamino-3-methyluracil (AFMU) (HMDB0011105), 1,3 or 3,7 dimethyluric acid (HMDB0001857, HMDB0001982), caffeine (HMDB0001847)	FFQ	[38]
Coffee	502	Serum	LC-MS, GC-MS	No	Trigonelline (HMDB0000875), quinic acid (HMDB0003072), 1-methylxanthine (HMDB0010738), paraxanthine (HMDB0001860), N-2-furoylglycine (HMDB0000439), catechol sulfate (not in HMDB)	FFQ	[39]
Coffee	498	Serum	LC-MS	No	Trigonelline (HMDB0000875), quinic acid (HMDB0003072), paraxanthine (HMDB0001860), N-2-furoylglycine (HMDB0000439), catechol sulfate (not in HMDB), caffeine (HMDB0001847), 1-methylxanthine (HMDB0010738), theophylline (HMDB0001889), trimethyluric acid (HMDB002123), hydroxyhippuric acid (HMDB0006116), 1,7-dimethyluric acid (HMDB0011103), 1-methyluric acid (HMDB0003099), cyclo(leu-pro) (HMDB0034276), 4-vinylphenol sulfate (HMDB0062775), hydroxyphenylpropionate (HMDB0000375), theobromine (HMDB0002825), cinnamoylglycine (HMDB0011621)	FFQ	[10]
Coffee	475	Urine (24 h)	LC-MS	No	Dihydroferulic acid sulfate (HMDB0041724), guaiacol glucuronide (not in HMDB), feruoylquinic acid (HMDB0030669), ferulic acid sulfate (HMDB0029200), feruoylquinic acid glucuronide (not in HMDB), 3-caffeoylquinic acid (HMDB0003164), p-coumaric acid sulfate (not in HMDB), caffeic acid sulfate (HMDB0041706), ferulic acid glucuronide (HMDB0041733), hydroxyhippuric acid (HMDB0006116), dihydrocaffeic acid sulfate (HMDB0041721), m-coumaric acid sulfate (not in HMDB), dihydroferulic acid glucuronide (HMDB0041723), p-hydroxyphenyllactic acid (HMDB0000755), guaiacol sulfate (not in HMDB), ethylcatechol glucuronide (not in HMDB)	24-HDR	[37]

Later, metabolomic studies found novel markers of coffee intake in observational studies using untargeted approaches. In a first such study, *N*-methylpyridinium and trigonelline, products of the coffee roasting process, were found to best distinguish coffee drinkers from non-coffee drinkers (after analysis of urine by LC-MS) [35]. Both compounds remained elevated in urine for at least 2 days after coffee consumption and were thus proposed as stable biomarkers of intake. *N*-methylnicotinamide also distinguished the two groups, although it was not considered specific to coffee intake, being a metabolite of niacin found in a wide range of foods. In another study, dihydrocaffeic acid and its 3-glucuronide, measured in 24-h urine by LC-MS, was found to discriminate groups of high- and low-coffee consumers (identified with a food frequency questionnaire) with high sensitivity and specificity [36]. A later cross-sectional study in 481 subjects of the EPIC cohort, also using 24-h urine samples, showed significant correlations between concentrations of 16 phenolic acids, mostly glucuronide or sulfate esters, with acute coffee intake as estimated with 24-h dietary recalls [37]. Dihydroferulic acid sulfate concentrations correlated most strongly with coffee intake whether assessed by FFQ (*r* = 0.62) or 24-h dietary recall (*r* = 0.52). Dihydroferulic acid sulfate, feruloylquinic acid glucuronide, ferulic acid sulfate, and guaiacol glucuronide were the metabolites whose measured intensities best classified subjects into the highest or lowest quintiles of coffee intake, with a receiver operating characteristic (ROC) area under the curve (AUC) for the predictive model > 94%. Non-phenolic metabolites were not investigated in this study.

The use of 24-h urine samples yields the widest range of potential biomarkers, as all coffee metabolites accumulate in urine after coffee intake regardless of metabolite pharmacokinetics. Biomarkers identified in 24-h urine samples cannot be assumed to be effective markers when measured in spot urine or in blood samples taken at a single time point since many food-derived metabolites are excreted rapidly after absorption. A few authors have searched for markers of coffee intake in spot urine or blood collections (Table 2). For example, biomarkers of coffee intake were searched for in morning spot urines of French subjects from the SUVIMAX cross-sectional study [38]. The intensities of several coffee-derived metabolites accurately classified consumers into high- and low-intake groups (respectively 183–540 and vs. 0 mL/day, as measured with repeated 24-h dietary recalls and a food frequency questionnaire). The most effective of these were the diterpene atractyligenin glucuronide (*r* = 0.534, ROC AUC = 0.98), the cyclic amino acid cyclo(isoleucylprolyl) (*r* = 0.543, ROC AUC = 0.969) and the caffeine metabolite 1-methylxanthine (*r* = 0.561, ROC AUC = 0.965). Also, urinary concentrations of 1,7-dimethyluric acid, 1-methyluric acid, and trigonelline each classified subjects with an excellent sensitivity and specificity (ROC AUC > 0.9). Combining cyclo(isoleucylprolyl), 1-methylxanthine, and trigonelline concentrations as a single biomarker increased classification performance relative to any one single compound. Hippuric acid was elevated in the urine of coffee consumers, while caffeoylquinic acid-derived metabolites were not reported as discriminants in this study.

Two further studies have identified biomarkers of coffee intake in blood. Trigonelline, 1-methylxanthine, and paraxanthine were identified as serum biomarkers of coffee intake when comparing high- (> 2.5 cups/day) and low- (< 2.5 cups/day) coffee drinkers in an American-nested case-control study, along with *N*-2-furoylglycine and catechol sulfate [39]. A more detailed study on coffee in the same cohort additionally reported that plasma trigonelline (partial *r* = 0.608) and quinic acid (partial *r* = 0.59) concentrations best correlated with coffee intake as reported by FFQ [10]. In contrast, concentrations of unmetabolized caffeine correlated moderately with coffee intake (partial *r* = 0.327).

The studies described above proposed biomarkers of intake exclusively in urine, plasma, or serum. A small number of studies have considered coffee-derived metabolites in other biospecimens. For example, after a dose of coffee, a Japanese group was able to measure caffeine and three isomers of dimethylxanthine in fingerprints [40]. Also, pyridine was found to increase in breath after consumption of a large cup of espresso [41]. Such techniques may not be applicable to epidemiological studies at present but represent possible future alternatives to measuring biomarkers of coffee intake in blood and urine.

In summary, many exogenous blood and urinary metabolites have been proposed as coffee intake markers but their validity depends on study design, study population, biofluid, and analytical method. In intervention studies where urine or blood samples are taken shortly after the administration of coffee, hydroxycinnamic acid derivatives such as caffeic and dihydroferulic acid (and their phase II conjugates) have been most commonly proposed as biomarkers of coffee intake. Most recent studies in free-living subjects suggest that several phenolic acids (ferulic, isoferulic, dihydroferulic, caffeic, and dihydrocaffeic acids and their glucuronides and sulfate esters), alkaloids (caffeine, trigonelline, and their metabolites), cyclo(isoleucylprolyl), and atractyligenin glucuronide, measured in urine, are the most sensitive and specific biomarkers of coffee intake. Fewer studies in free-living subjects were conducted on blood and trigonelline, and quinic acid were found to best correlate with coffee intake.

Still, it will be important to check the specificity of these biomarkers in the populations where implemented as the levels of coffee intake and the impact of possible confounders may vary between populations. Indeed, some of these biomarkers like caffeine or ferulic acid may also arise from other dietary sources such as tea or soft drinks for caffeine or wholegrain cereals for ferulic acid. Trigonelline and cyclo(isoleucylprolyl) or their precursors may be preferred considering their high specificity for coffee.

### Tea biomarkers

#### Tea metabolites in controlled intervention studies

Metabolism and pharmacokinetics of catechins have been studied in a large number of intervention studies with green or black tea (Table 3). The main compounds detected in biofluids after green tea ingestion are catechins, and their metabolites formed in phase II biotransformations (methylation, glucuronidation, and sulfation) and ring-fission reactions. EGCG, EGC, ECG, and EC were the main compounds detected in plasma. They are quickly absorbed, and peak concentrations are observed about 2 h after ingestion [42]. Tea catechins are also quickly excreted, and their elimination half-lives usually do not exceed 3 h. EGC is the most abundant catechin in plasma after tea intake, mainly found in its glucuronidated form [42]. EGC is also methylated in the liver, and 4′-*O*-methyl-EGC is found in both sulfated and glucuronidated forms. Galloylated catechins (EGCG and ECG) are present in plasma in their non-conjugated forms.

**Table 3:** Metabolites identified in human intervention studies on tea

Type of beverage	No. subjects	Sample type	Analytical method	Enzymatic hydrolysis	Discriminating metabolites/candidate biomarkers	Reference
Green tea, black tea	18	Urine	LC-ECD	Yes	Epicatechin, epigallocatechin	[49]
Green tea, black tea	20	Urine	GC-MS	Yes	4-O-Methylgallic acid	[128]
Black tea	10	Plasma, urine	HPLC	?	4-*O*-Methylgallic acid, gallic acid	[55]
Green tea, black tea (extracts)	17	Urine	LC-MS	No	Hippuric acid	[129]
Green tea, black tea (decaffeinated)	133	Urine	LC-ECD	Yes	(−)-Epigallocatechin	[60]
Green tea, black tea	30	Plasma	LC-ECD	Yes	(−)-Epicatechin, (−)-epicatechin-3-gallate, (−)-epigallocatechin, (−)-epigallocatechin-3-gallate	[61]
Green tea	10	Plasma, urine	LC-MS	No	(−)-Epicatechin-3’-O-glucuronide, (epi)catechin-O-sulfates, 3’-O-methyl-(epi)catechin-O-sulfates, 4’-O-methyl-(epi)catechin-O-sulfate, (epi)gallocatechin-O-glucuronide, 4’-O-methyl-(epi)gallocatechin-O-sulfates (urine and plasma); (−)-epicatechin-3-O-gallate, 4’-O-methyl-(epi)gallocatechin-O-glucuronide, (−)-epigallocatechin-3-O-gallate (plasma only); (epi)gallocatechin-O-sulfates (urine only)	[42]
Green tea	20	Urine	LC-MS	No	(Epi)catechin glucuronide, (epi)catechin sulfate, (epi)catechin sulfoglucuronide, methyl(epi)catechin sulfate, (epi)gallocatechin glucuronide,(epi)gallocatechin sulfate, methyl(epi)gallocatechin glucuronide, methyl(epi)gallocatechin sulfate, methyl(epi)gallocatechin sulfoglucuronide, 5-(hydroxyphenyl)-γ-valerolactone glucuronide, 5-(hydroxyphenyl)-γ-valerolactone glucuronide sulfate, 5-(3′,5′-dihydroxyphenyl)-γ-valerolactone glucuronide,5-(3′,5′-dihydroxyphenyl)-γ-valerolactone glucuronide disulfate, 5-(4′,5′-dihydroxyphenyl)-γ-valerolactone glucuronide, 5-(4′,5′-dihydroxyphenyl)-γ-valerolactone glucuronide disulfate, 5-(4′,5′-dihydroxyphenyl)-γ-valerolactone glucuronide sulfoglucuronide, methyl-5-(4′,5′-dihydroxyphenyl)-γ-valerolactone glucuronide, 5-(3′,4′,5′-trihydroxyphenyl)-γ-valerolactone glucuronide, 5-(3′,4′,5′-trihydroxyphenyl)-γ-valerolactone glucuronide sulfate, methyl-5-(3′,4′,5′-trihydroxyphenyl)-γ-valerolactone glucuronide, methyl-5-(3′,4′,5′-trihydroxyphenyl)-γ-valerolactone sulfate	[43]
Black tea	4	Urine	LC-MS	No	(Epi)catechin sulfate, O-methylcatechin sulfate, O-methyl(epi)catechin sulfates, O-methyl(epi)gallocatechin sulfates, di-O-methyl(epi)gallocatechin sulfates, dihydronaringenin sulfates, 3’-O-methyl-5-(3′,4′-dihydroxyphenyl)-γ-valerolactone 4’-O-glucuronide, 4’-O-methyl-5-(3′,4′-dihydroxyphenyl)-γ-valerolactone 3’-O-glucuronide, 5-(3′,4′,5′-trihydroxyphenyl)-γ-valerolactone 3’-O-glucuronide, 4’-O-glucuronide, 3’-O-sulfate, 4’-O-sulfate and sulfoglucuronide, O-methyl-5-(3′,4′,5′-trihydroxyphenyl)-γ-valerolactone 3′- or 4’-O-glucuronides, 3′ or 5’-O-glucuronide and O-sulfate, 5-(3′,4′-dihydroxyphenyl)-γ-valerolactone 3’-O-glucuronide, 4’-O-glucuronide and sulfates, 5-(3′,5′-dihydroxyphenyl)-γ-valerolactone 3’-O-glucuronide and 3’-O-sulfate, O-methyl-5-(3′,5′-dihydroxyphenyl)-γ-valerolactone glucuronides and sulfates, 5-(3′-hydroxyphenyl)-γ-valerolactone 3’-O-glucuronide, 4’-O-glucuronide, 5-(hydroxyphenyl)-γ-valerolactone sulfate, O-methyl-4-hydroxy-5-(3′,4′,5′-trihydroxyphenyl)valeric acid glucuronides, 4-hydroxy-5-(3′,4′-dihydroxyphenyl)valeric acid glucuronides, O-methyl-4-hydroxy-5-(3′,4′-dihydroxyphenyl)valeric acid glucuronides, 4-hydroxy-5-(3′,5′-dihydroxyphenyl)valeric acid glucuronide, O-methyl-4-hydroxy-5-(3′,5′-dihydroxyphenyl)valeric acid glucuronides, 4-hydroxy-5-(3′-hydroxyphenyl)valeric acid 3’-O-sulfate and 4’-O-sulfate, O-methyl-4-hydroxy-5-(dihydroxyphenyl)valeric acid sulfates, 4-hydroxy-5-(dihydroxyphenyl)valeric acid sulfates, O-methyl-4-hydroxy-5-(hydroxyphenyl)valeric acid sulfate, 4-hydroxy-5-(phenyl)valeric acid glucuronides and sulfates, hippuric acid, indole-3-acetic-acid glucuronide, indole-3-carboxylic acid glucuronide, p-cresol sulfate and glucuronide, pyrogallol 2-O-glucuronide, 1-O-sulfate and 2-O-sulfate, urolithin A-3-O-glucuronide, 8-O-glucuronide and sulfoglucuronide, urolithin B-O-glucuronide, vanilloylglycine, vanillic acid-4-O-glucuronide and 4-O-sulfate, phenylacetylglycine	[130]
Green tea (extract)	14	Urine	LC-MS	Yes	Catechin,, epicatechin, 3’-O-methylepicatechin, 4’-O-methylepicatechin, epicatechin-3-O-gallate, gallocatechin, gallocatechin gallate, epigallocatechin, 3’-O-methylepigallocatechin, 4’-O-methylepigallocatechin, epigallocatechin-3-O-gallate, 5-(3′,4′,5′-trihydroxyphenyl)-γ-valerolactone, 5-(3′,4′-dihydroxyphenyl)valerolactone, 5-(3′,5′-dihydroxyphenyl)valerolactone, gallic acid, 3-O-methyl gallic acid, 3-hydroxybenzoic acid, syringic acid, benzoic acid, hippuric acid	[131]
Black tea (extract)	12	Plasma	LC-MS	No	(Epi)catechin sulfate, O-methyl(epi)catechin sulfates, di-O-methyl(epi)catechin sulfate, O-methyl(epi)gallocatechin sulfate, (epi)catechin gallate sulfate and sulfoglucuronide, O-methyl(epi)catechin gallate sulfate and sulfoglucuronide, (epi)gallocatechin gallate, (epi)gallocatechin gallate sulfate, O-methyl(epi)gallocatechin gallate sulfate, δ-(3′,4′-dihydroxyphenyl)-γ-valerolactone) 3′-O-glucuronide, 4′-O-glucuronide, 3′-O-sulfate and sulfoglucuronide, 5-(3′-hydroxyphenyl)-γ-valerolactone 3′-O-glucuronide and 3′-O-sulfate, 5-(4′-hydroxyphenyl)-γ-valerolactone 4′-O-glucuronide, 5-(3′,4′,5′-trihydroxyphenyl)-γ-valerolactone 3′-O-glucuronide, 4′-O-glucuronide, 3′-O-sulfate and sulfoglucuronide, O-methyl-5-(3′,4′,5′-trihydroxyphenyl)-γ-valerolactone 3′/4′-O-glucuronide, 3′/5′-O-glucuronide and O-sulfates, 5-(3′,5′-dihydroxyphenyl)-γ-valerolactone 3′-O-glucuronide and 3′-O-sulfate, O-methyl-5-(3′,5′-dihydroxyphenyl)-γ-valerolactone 3′-O-glucuronide and 3′-O-sulfate, 4-hydroxy-5-(dihydroxyphenyl)valeric acid sulfates, O-methyl-4-hydroxy-5-(hydroxyphenyl)valeric acid sulfates, 4-hydroxy-5-(3′,4′-dihydroxyphenyl)valeric acid glucuronides, 4-hydroxy-5-(3′,5′-dihydroxyphenyl)valeric acid glucuronide, O-methyl-4-hydroxy-5-(3′,5′-dihydroxyphenyl)valeric acid glucuronide, O-methyl-4-hydroxy-5-(3′,4′,5′-trihydroxyphenyl)valeric acid glucuronide, kaempferol glucuronide, O-methylgallic acid sulfates, pyrogallol 2-O-glucuronide and 2-O-sulfate, O-methylcatechol sulfates, resorcinol glucuronide and sulfate, hippuric acid	[54]
Black tea (extract)	19	Plasma	LC-MS	Yes	(−)-Catechin, (−)-epicatechin, (−)-(epi)catechin gallate, (−)-epigallocatechin, (−)-epigallocatechin gallate, isorhamnetin, 3/4-O-methylgallic acid, 5-(3′,4′-dihydroxyphenyl)-γ-valerolactone, 5-(3′-methoxy-4′-hydroxyphenyl)-γ-valerolactone	[56]
Black tea (extract)	12	Plasma	GC-MS	Yes	4-O-Methylgallic acid, gallic acid, hippuric acid, pyrogallol	[57]
Green tea, black tea	93	Urine	LC-ECD	Yes	(−)-Epicatechin, (−)-epigallocatechin, 4’-O-methylepigallocatechin	[62]

Catechins not absorbed in the small intestine reach the colon where they can be degraded by the microbiota into low-molecular weight metabolites such as hydroxyphenylvalerolactones, hydroxyphenylvaleric acids, phenolic acids, and hippuric acid. These metabolites show longer elimination half-lives in urine where they persist for 48 h after tea intake [43].

Most studies showed a linear relationship between plasma concentrations or urine excretion of tea catechins and the ingested dose [44–48] although concentrations of some catechins were also shown to reach a plateau at a high level of intake in some studies [49, 50].

Many untargeted metabolomic studies have been conducted to elucidate additional candidate biomarkers of tea intake [51–53]. In a placebo-controlled cross-over intervention study based on high-resolution mass spectrometry, 12 male subjects consumed a single capsule of tea extract (2.65 g) or a placebo after 1 day of a polyphenol-poor diet [54]. Fifty-nine polyphenol metabolites were identified and increased in concentration after black tea ingestion. These metabolites were catechins, phenolic acids, valerolactones, and simple phenols, most in the form of glucuronides and sulfate esters. The highest concentrations in plasma were observed within 1–4 h (catechins, kaempferol, gallic acid) or 5–10 h (microbial metabolites such as phenylvalerolactones, pyrogallol, and hippuric acid), suggesting that they would be better indicators of tea intake if measured in 24-h urine samples rather than spot urine samples.

Some markers may be used to differentiate intake of green and black tea. In particular, 4-*O*-methylgallic acid, formed by *O*-methylation of gallic acid, has frequently been reported in both urine and plasma after black tea intake [55–57]. This metabolite is also detected in urine after green tea intake but in much lower concentrations [58]. This difference in concentrations is explained by the low content of gallic acid in green tea when compared to black tea. Measurement of 4-*O*-methylgallic acid in urine or plasma could thus be useful to differentiate consumption of black and green tea, particularly if used in combination with catechins more abundant in green tea than in black tea [59] and present at higher concentrations in plasma or urine after consumption of green tea when compared to black tea [49, 60–62]. Theaflavins and thearubigins are abundant in black tea and absent from green tea; however, their high molecular weight considerably limits or prevents their absorption in the gut [63] and they therefore cannot be used as biomarkers of black tea intake.

Catechin metabolites formed by the gut microbiota are, in comparison with catechins or gallic acid, less useful as biomarkers of tea intake. Some of these metabolites (some phenolic acids and hippuric acid) were present in plasma in the absence of tea consumption [54]. This is explained by the existence of precursors of these metabolites in other dietary sources such as coffee, cocoa, fruits, or vegetables beside tea [64–66]. Other metabolites derived from tea catechins such as 5-(3′,4′,5′-trihydroxyphenyl)-γ-valerolactone, 5-(3′,4′-dihydroxyphenyl)- γ-valerolactone, and/or 5-(3′,5′-dihydroxyphenyl)- γ-valerolactone may be more specific for tea intake [48, 67]. However, formation of these metabolites from catechins shows high interindividual variations, compromising their potential use as biomarkers of intake [68]. Similarly, other tea constituents such as quercetin or caffeine may also be of limited utility as biomarkers due to their abundance in other foods and beverages [69, 70].

#### Biomarkers of tea intake in observational studies

As previously highlighted, observational studies allow the investigation of biomarker sensitivity and specificity. In the Shanghai Cohort Study, EGC, 4′-*O*-methyl-EGC, EC, and two phenylvalerolactones measured in urine were significantly associated with self-reported tea intake [71, 72]. In another study carried out in 481 subjects from the EPIC cohort, metabolic profiles were compared in 24-h pooled urine samples by high-resolution MS in tea consumers and non-consumers (identified with 24-h dietary recalls) [37]. Several phenolic compounds were found to be associated with recent tea intake as assessed with a 24-h dietary recall. The compound that best distinguished non- and high consumers was found to be 4-*O-*methylgallic acid (ROC AUC = 0.84). 4-*O-*Methylgallic acid was also associated with habitual tea intake as assessed with a FFQ despite its short-elimination half-life, and this was explained by frequent tea intake in this population. However, some confounding with red wine intake was also observed. Other polyphenol metabolites were also found to be associated with tea intake in the same study (methyl(epi)catechin sulfate, dihydroxyphenyl-γ-valerolactone sulfate, hydroxyphenylvaleric acid glucuronide, and pyrogallol sulfate), but they may not be more specific for tea than 4-*O-*methylgallic acid, as other dietary sources of the same polyphenol metabolites are also known. Excretion of catechin metabolites in urine were also found to be correlated with intake of chocolate products, apples, and pears. Pyrogallol sulfate has also been identified in plasma after intake of nuts [73] or mixed berry fruit [74]. In another cross-sectional study, 24-h urinary kaempferol was correlated with tea intake (*r* = 0.41; *p* < 0.01) [75], but kaempferol was also found to be correlated with onion in another study [76]. Therefore, most of these markers associated with tea intake may not be specific enough for tea, depending on the possible consumption of confounding foods in the population considered.

Some catechins might be the most specific biomarkers for tea intake. Three catechins, EGCG, EGC, and ECG, are mainly or exclusively found in tea according to the Phenol-Explorer database [59]. Galloylated catechins EGCG and ECG and their methylated metabolites have been detected in both plasma and urine in tea intervention studies, but they may be more difficult to measure in population studies due their limited absorption in the gut when compared with other catechins [77, 78]. In summary, EGC and its phase II derivative 4′-*O*-methyl-EGC have been measured in several cohort studies and might be a useful biomarker for green and black tea intake [71, 72]. 4-*O*-Methylgallic acid, a metabolite of gallic acid (particularly abundant in black tea), has been associated with black tea consumption in both intervention and observational studies (Tables 3 and 4). Ratio of 4-*O*-methylgallic acid over EGC is expected to be higher in black tea consumers when compared to green tea consumers and could help to discern which of the two types of tea has been consumed. A similar approach using ratios of specific alkylresorcinols was used for discriminating between wholegrain wheat and rye intakes [79, 80]. Other constituent characteristic of tea like theanine have not received much attention and should also be tested as possible biomarkers of tea intake. Biomarkers of tea intake are summarized in Table 4.

**Table 4:** Biomarkers of tea intake discovered in observational studies

Type of beverage	No. subjects	Sample type	Analytical method	Enzymatic hydrolysis	Discriminating metabolites/candidate biomarkers (HMDB ID if available)	Association with	Reference
Black tea	232	Urine (24 h)	GC-MS	Yes	4-*O*-Methylgallic acid (HMDB0013198)	24-HDR	[132]
Black tea	53	Urine (24 h and spot)	LC-MS	Yes	Gallic acid (HMDB0005807), 4-O-methylgallic acid (HMDB0013198)	2-Day dietary record	[33]
Green tea	968	Urine	LC-MS	Yes	Epicatechin (HMDB0001871), (−)-epigallocatechin (HMDB0038361), 4’-O-methylepigallocatechin (not in HMDB), 5-(3′,4′,5′-trihydroxyphenyl)-γ-valerolactone (HMDB0041691), 5-(3′,4′-dihydroxyphenyl)-γ-valerolactone (HMDB0029185)	FFQ	[71]
Green tea, black tea	119	Urine (24 h)	LC-MS	Yes	Kaempferol (HMDB0005801)	FFQ, 4-day food diary	[75]
Green tea	660	Urine (spot, non-fasting)	HPLC	Yes	Epicatechin (HMDB0001871), (−)-epigallocatechin (HMDB0038361), 4’-O-methylepigallocatechin (not in HMDB), 5-(3′,4′,5′-trihydroxyphenyl)-γ-valerolactone (HMDB0041691), 5-(3′,4′-dihydroxyphenyl)-γ-valerolactone (HMDB0029185)	FFQ	[72]
Tea	191	Urine (24 h, overnight)	LC-MS	Yes	Kaempferol (HMDB0005801)	FFQ	[133]
Tea	476	Urine (24 h)	LC-MS	No	Methyl(epi)catechin sulfate (not in HMDB), hydroxyphenylvaleric acid glucuronide (not in HMDB), dihydroxyphenyl-γ-valerolactone glucuronide and sulfate (HMDB0041693, HMDB0029191), 4-O-methylgallic acid (HMDB0013198), methylgallic acid sulfate(HMDB0060005), pyrogallol sulfate (HMDB0060016)	24-HDR	[37]

### Sugar-sweetened beverage biomarkers

Identification and validation of markers reflecting the consumption of SSBs are an important task to better assess the association between the consumption of SSBs and related health effects. Major challenges to identify such biomarkers are linked to the broad definition of what a SSB is and to the variability of their composition. One major point is the nature of the caloric sweeteners used. Beverages containing added caloric sweeteners such as sucrose or high-fructose corn syrup (HFCS) are the main types of SSB. These beverages form a very heterogeneous group comprising soft drinks, fruit drinks, sports drinks, energy drinks, flavored water drinks, and iced teas [81–83]. Given this diversity of products and their different composition, having specific markers that reflect the intake of individual products or the total daily consumption of SSBs is challenging.

The dominating ingredient in all SSBs is, by definition, the added sugar. Exposure markers for the intake of sugar have been described using two different approaches, either through the measurement of the carbon isotope ratio ^13^C/^12^C (expressed as δ^13^C value) or through the determination of sugars in urine. The first approach is based on the different discrimination against carbon dioxide formed from the ^13^C and ^12^C isotopes in plants. Crop species have been classified as C3 and C4 plants depending on their photosynthetic pathway. The photosynthetic pathway of C3 plants like sugar beet discriminates against ^13^CO_2_ compared with ^12^CO_2_, and thus, the resulting plant mass carbon has a lower ^13^C/^12^C ratio than atmospheric CO_2_. In contrast, the C4 pathway is almost non-discriminating against ^13^C, resulting in a plant mass higher in ^13^C compared to C3 plants. Sugarcane and corn, the main sources for sugar production in the USA, are C4 crops. As a consequence, sugar from these crops is enriched in ^13^C, compared to sugar produced by C3 plants. This enrichment can be seen in whole human biospecimens or specific metabolites in these biospecimens after consumption of sugars from C4 plants.

The ^13^C/^12^C isotope ratio of blood plasma, finger-prick blood, or of the amino acid alanine either from hair protein or red blood cells have been proposed in various studies to predict intake of SSBs and added sugar in the USA (Table 5) [84–88]. However, this approach also has limitations depending on the nature of the sugar sources consumed in various populations. While in the USA, added sugar is mostly derived (78%) from sugarcane or corn [89], the situation in Europe is the opposite with around 80% added sugar derived from the C3 plant sugar beet [90]. In consequence, the use of ^13^C as a potential exposure marker for added sugar or SSBs is limited to the USA. Additionally, ^13^C in whole blood is also influenced by the intake of corn products and meat from livestock fed on corn, and thus, the intake assessment of sugar might be confounded. Two methods have been described to correct for these confounding food items. The first method uses nitrogen-15 which is elevated in marine foods as well as in meat products. Therefore, it was suggested to use this second isotopic marker as control for the intake of animal protein [86, 87, 91, 92]. Nash et al. [87, 92] found favorable results in a study population of Yup’ik Eskimos consuming low amounts of sugar from sources not ^13^C-enriched such as sugar beet, honey, fruits, or dairy products, but high amounts of fish and marine mammals compared to other US populations. They were able to explain three times as much variation in the sweetener intake by using a model including both the carbon and nitrogen isotope ratios than by using δ^13^C alone. In contrast, Fakhouri et al. [86] and Hedrick et al. [91] found no significant improvement in their results after correcting for the animal protein intake using nitrogen-15 in a population where more corn-fed meat is consumed. The second method to correct for confounders is based on the use of a specific metabolite to measure ^13^C, which favors the incorporation of glucose carbon like alanine as described by Choy et al. [84]. They found no association between the ^13^C of alanine and dietary confounders like commercial meat, fish, and corn products. At the same time, they found that a dual-isotope model using ^13^C and ^15^N in red blood cells was associated with meat intake as well as sweeteners. The use of alanine as a specific metabolite shows favorable results in this respect, but further research is needed, especially concerning different populations.

**Table 5:** Biomarkers of intake for sugar-sweetened beverages discovered in observational studies

No. subjects	Analytical method	Sample type	Discriminating metabolites/candidate biomarkers (HMDB ID)	Association with	Reference
144	IRMS	Serum	δ^13^C	24-HDR (× 2)	[86]
60	IRMS	Blood	δ^13^C	4-day dietary record	[85]
68	GC-IRMS	Red blood cells	δ^13^C of alanine (HMDB0000161)	24-HDR (× 2)	[84]
68	IRMS	Red blood cells, plasma, hair	δ^13^C	24-HDR	[87]
68	IRMS	Fasting plasma	δ^13^C of glucose (HMDB0000122)	24-HDR	[87]
257	IRMS	Blood	δ^13^C	24-HDR (× 3), 4-day dietary record	[91]
565	^1^H-NMR	Urine	Formate (HMDB0000142), citrulline (HMDB0000904), taurine HMDB0000251, isocitrate (HMDB0000193)	4-day dietary record	[95]

The second approach for calculating sugar intake uses urinary sucrose and fructose as exposure markers [93, 94]. Details on the different intervention studies and surveys were recently reviewed [93]. For this exposure marker, no information on its applicability for SSBs is currently available.

Both approaches, the carbon isotope ratio of different biospecimens and urinary fructose and sucrose, show promise as exposure markers for sugar and sucrose intake. However, sugar alone does not appear suitable as an exposure marker for SSB consumption because it is also contained in hundreds of other food items and is thus not specific. The use of an exposure marker for sugars in combination with additional substances used as ingredients might prove to be more specific. However, ingredients used in SSBs vary depending upon the type of SSB. Citric acid is added to many types of widely distributed SSBs as acidity regulator. However, citric acid seems not suitable as a marker for SSB consumption since it is produced in large quantities in the human metabolism. Moreover, it also naturally occurs in large concentrations in fruits and fruit juices. Other typical ingredients in SSBs are natural or artificial flavors such as ginger extract for ginger ale or caffeine for cola type beverages. Using these compounds or their metabolites as markers would consequently only cover specific subgroups of SSBs. Their specificity should also be carefully assessed as some of these substances may also be ingested with other foods, like coffee in case of caffeine.

A more reliable approach to reach a high specificity when assessing intake of a whole food group such as SSBs might be to use a combination of exposure markers as described by Gibbons et al. [95]. In this study, a subcohort of 565 participants from the National Adult Nutrition Survey was divided into four quartiles. Four metabolites detected by NMR spectroscopy in urine—formate, citrulline, taurine, and isocitrate—showed concentration levels that differed significantly between SSB consumers (*n* = 146) and non-consumers (*n* = 391) as identified via a 4-day semi-weighed food record. Levels of these same four metabolites also showed transient and modest increase in an acute intervention study (*n* = 10) after the consumption of one can of cola. The authors detected all four markers in the cola drink used for the acute intervention study. Although found in the cola drink, none of these compounds are normally added in a pure form so that they would most likely originate from added flavor extracts. It also remains questionable whether these markers are indicative of intake of the whole group of SSBs or cola consumption only. So-called energy drinks often contain taurine, a sulfur-containing amino acid. Thus, taurine might serve as a marker for this specific type of beverage. However, it is unlikely that taurine is present in all types of SSB. Furthermore, other sources of taurine cannot be excluded. Taurine is formed in human metabolism from methionine and cysteine with an endogenous average daily synthesis of 0.4–1.0 mmol (50–125 mg) [96]. Meat and seafood are significant dietary sources of this amino acid [93]. Similarly, isocitrate is an intermediate of the citric cycle—like citrate—and, therefore, occurs in all humans, animals, and plants. These examples demonstrate that the urinary excretion of these compounds can have different origins that interfere with their use as markers of exposure for SSBs. Further validation of the proposed panel of biomarkers in other populations is still necessary. Furthermore, more research is needed to identify new markers or marker panels with higher specificity and selectivity.

In summary, biomarkers of sugar intake would not be ideal biomarkers of SSB intake due the diversity of dietary sources of sugar. One exception may be the ^13^C/^12^C isotope ratio in countries where sugarcane and corn sugar is more specifically used as SSB sugar. Other SSB ingredients or combinations of ingredients might be used as biomarkers of intake for SSBs or for specific types of SSB. However, none of these biomarkers have yet been validated in population studies.

### Low-calorie-sweetened beverage biomarkers

No studies were identified which specifically aimed to identify biomarkers of LCSB intake. Rather, many of the studies investigated the impact of LCSB consumption on health via the measurement of effect biomarkers in relation to metabolic syndrome [97], blood pressure [98], and glycemic control [99, 100]. LCSB consumption was found to influence the concentration of various compounds including urinary hippuric acid excretion [101], serum uric acid concentrations [102], and plasma free fatty acid concentrations [103]. However, these biomarkers are not specific to LCSBs; hippuric acid is a potential marker of toluene exposure, as well as fruit and vegetable intake, and serum uric acid and plasma free fatty acids are endogenous compounds influenced by factors other than LCSB intake. Therefore, utilization of these biomarkers is unlikely to provide useful information on LCSB intake owing to a lack of specificity. Several advanced glycation end products were found to be present in regular and, to a lesser extent, in diet cola drinks. They are normally excreted via the urine, but they are also confounded by other more significant dietary sources of these compounds [104]. Brominated vegetable oil (BVO) is used in North America as a clouding agent in some soft drinks but is not permitted for use in many other areas, including the European Union, owing to concerns about bromine toxicity. Bendig et al. assessed the BVO content of commonly consumed soft drinks, and BVO was detected in only three out of the ten drinks investigated indicating that tissue bromine concentrations would not serve as a reliable biomarker of LCSB intake [105]. Eisner et al. assessed the citrate and malate content of diet beverages in the context of the treatment of nephrolithiasis (kidney stones) [106], but these two organic acids are not specific for LCSBs and may be directly influenced by other dietary and non-dietary factors or may not be relevant for all LCSBs.

Given that the term LCSB encompasses an array of products including carbonated beverages, fruit cordials, sweetened dairy products, and flavored teas, all of which are likely to differ significantly in their composition, the identification of a specific biomarker of intake which is specific to all LCSBs as a single food group is likely to be challenging. The low-calorie sweeteners (LCSs) themselves, common to a large diversity of LCSBs, may prove to be the most useful biomarkers of LCSB intake. There are eleven LCSs currently approved for use on the European market (Additional file 2: Table S1), and their use extends to a wide range of foods other than LCSBs, as well as non-dietary products such as oral hygiene products and e-cigarette fluids [107]. Although this may complicate the use of LCSs as valid markers of LCSB intake, LCSBs are widely considered to be the primary source of LCSs in the diet; and therefore, measurement of these as biomarkers may provide a viable option for assessing LCSB intake.

The potential application of a biomarker approach for investigating intakes of low-calorie sweeteners (LCS) has recently been reviewed [107]. The metabolic fate of each LCS is assessed prior to approval for use as a food additive (Table 6); and therefore, a targeted approach may be implemented for those that lend themselves well to analyses. A number of LCSs are excreted in urine in the same form as found in the LCSBs. Acesulfame-K [108] and saccharin [109–111] are almost completely absorbed and excreted unchanged via the urine. The usefulness of urinary excretions of these two LCSs as biomarkers of intake was investigated by Wilson et al. who measured levels of excretion in 24-h urine samples and found high correlation with acute intakes of saccharin and acesulfame-K in an intervention study with five different doses of the LCS (*R*^2^ > 0.99 for both compounds), demonstrating a clear dose-response relationship for both compounds [112]. Two other LCSs, cyclamate [113] and sucralose [114, 115], are partially absorbed (respectively 30–50 and 10–15%) and subsequently excreted unchanged in urine. A proportion of cyclamate is converted to cyclohexylamine via bacterial hydrolysis in the gut in around 20% of the population. This is then absorbed and excreted via the urine [116]. The process of conversion to cyclohexylamine is highly variable between and within individuals, particularly during chronic exposure [113]; and therefore, some doubt must be cast on its usefulness as a reliable marker of cyclamate intake in the free-living population. A relatively new and increasingly used LCS, a mixture of steviol glycosides isolated from the leaf of *Stevia rebaudiana*, undergoes bacterial hydrolysis in the gut prior to absorption and subsequent excretion in urine as steviol glucuronide [117–119]. Advantame is deesterified to advantame acid and a small proportion is absorbed (~ 6%) and excreted via the urine [120]. The excretion products of acesulfame-K, saccharin, sucralose, cyclamates, steviol glycosides, and advantame are highly specific to exposures to the parent compound per se as they are not produced endogenously. Therefore, any detection of these in urine would indicate intake of the related sweeteners. However, further work would be required to assess their specificity as biomarkers of LCSB intake.

**Table 6:** Metabolic fates of low-calorie sweeteners approved in the European Union (adapted from [107])

Sweetener (CAS registry no.)	Metabolic fate	Route(s) of excretion^a^	References
Saccharin (81-07-2)	Not metabolised, excreted unchanged.	Urine	[109, 110]
Acesulfame-K (55589-62-3)	Not metabolised, excreted unchanged.	Urine	[108]
Aspartame (22839-47-0)	Hydrolysed to aspartic acid, phenylalanine and methanol.	N/A	[121, 122]
Cyclamate (139-05-9)	80% of the population do not metabolise cyclamate. In 20%, it undergoes partial hydrolysis in the gut to cyclohexylamine. Extent of hydrolysis varies between and within individuals.	Faeces, urine	[113]
Thaumatin (53850-34-3)	Undergoes normal protein digestion.	N/A	[123]
NHDC (20702-77-6)	Metabolised by gut microflora to similar metabolites to naturally occurring flavonoids.	Urine	[124]
Salt of aspartame-acesulfame (106372-55-8)	Dissociates to individual sweeteners in digestive fluids and undergoes same metabolic fates.	See information for acesulfame-K and aspartame	[134]
Sucralose (56038-13-2)	Not metabolised, excreted mainly unchanged but 2% of absorbed portion excreted as conjugates.	Faeces, urine	[114, 115]
Steviol glycosides^†^	Bacterial hydrolysis in the gut to steviol which is then absorbed and excreted as steviol glucuronide.	Urine	[117–119]
Advantam (714229-20-6)	Converted to advantame acid and mainly excreted as such with 2 minor metabolites.	Faeces, urine	[120]

*CAS* Chemical Abstract Service, *NHDC* neohesperidine dihydrochalcone, *N/A* not applicable as broken down to normal dietary components, *JECFA* Joint FAO/WHO Expert Committee on Food Additives

^a^Principal route of excretion listed

†No CAS registry, not available

Other LCSs, namely aspartame, neohesperidine dihydrochalcone (NHDC), and thaumatin, undergo extensive metabolism into metabolites commonly found in the diet or in the body, which suggests that no obvious specific biomarkers of intake exist for these LCSs. Aspartame is broken down to its three constituents, aspartic acid, phenylalanine, and methanol, all of which are often present in greater quantities in other dietary sources [121, 122]. Thaumatin, a naturally occurring protein complex, undergoes normal protein digestion; and therefore, identification of a specific biomarker of intake is not likely to be possible [123]. Finally, NHDC has a similar structure to naturally occurring flavonoids with similar metabolites; and for this reason, specificity of any putative biomarker is likely to be problematic [124].

In summary, a biomarker approach for assessing intake of specific LCSs used in LCSBs may prove useful as several of them are excreted unchanged in urine following ingestion. Expected biomarkers should be based on LCSs most commonly used in LCSBs. However, considering the range of LCSs used in different types of LCSBs, the geographical differences and the evolving nature of LCS use by manufacturers, further work is required to ensure the specificity of the putative biomarkers. This work may take the form of comprehensive and international label surveys along with biomarker validation studies for specific LCSBs. As such, more work will be needed to validate their use as biomarkers of LCSB intake, considering all these factors, and to identify possible confounding by other foods containing the same LCSs.

## Conclusions

A wide diversity of biomarkers has been proposed to estimate the consumption of non-alcoholic beverages. Metabolism of major constituents of non-alcoholic beverages has been studied in a large number of controlled intervention studies, and many metabolites were identified mainly in blood and urine (Tables 1 and 2). Participants in these studies have usually consumed a high amount of a particular beverage with a fully controlled diet after a proper washout period. These studies are useful for identification of putative biomarkers of intake, but provide limited evidence of their potential value as biomarkers of intake in a population and more particularly of their sensitivity and specificity.

Several of these candidate biomarkers have been further studied in observational studies with individuals following their own diet and their sensitivity and specificity as indicators of coffee, tea, and SSB intake evaluated (Tables 1, 2, and 3). Various compounds including phenolic acids, alkaloids, and terpenes measured in urine or plasma samples were shown to accurately predict coffee intake in various populations, and EGC and 4-*O-*methylgallic acid were also shown to be good indicators of tea intake. These two last biomarkers could also possibly be used to differentiate intake of green and black tea. Several biomarkers have been proposed to estimate SSB intake but none for LCSB intake. Difficulties met in finding biomarkers for these two last classes of beverages are explained by the lack of constituents that would be at the same time characteristic of each of these two groups of beverages and absent in all other foods.

Controlled intervention studies and observation studies are complementary. The first ones provide direct evidence of the causality of the associations between beverage intake and the biomarker and allow establishing the dose-effect relationship. Observational studies, although relying on self-reported estimate of beverage intake that are liable to errors, allow to study the sensitivity and selectivity of a biomarker and to identify potential confounders.

Combinations of biomarkers may be needed to assess intake of SSBs and LCSBs. Combinations of biomarkers may also allow the estimation of intake of different types of beverages within a particular group, as suggested for green and black tea. Combination of a generic biomarker for coffee with caffeine should help assess relative intake of caffeinated and decaffeinated coffee. Panels of biomarkers of intake have been proposed, but none has yet been validated.

The new biomarkers, identified through metabolomics or other approaches, will need to be validated in populations sharing similar lifestyle and diet to the ones where these biomarkers will be used. All possible confounders (e.g., foods containing the same biomarker or a precursor transformed into the biomarker in the body) will need to be carefully considered [37]. More extensive food composition data for these compounds, often scattered across a large number of publications and not easily analyzed, will have to be collected and made easily accessible in new databases such as those developed for polyphenols or for caffeine [59, 125]. Biomarkers with long elimination half-lives should be preferred particularly to assess intakes of foods more episodically consumed [126] although this may be less important for beverages like coffee or tea most often consumed on a daily basis [37]. Finally, the practicality of biomarkers will need to be assessed, including their performance according to the type of biospecimens (e.g., urine vs. blood, fasting vs. non-fasting blood samples or 24-h urine samples vs. spot urine samples), performance of analytical methods, and cost of analyses.

Overall, biomarkers should help estimate intake of non-alcoholic beverages and this may be particularly useful in overcoming some of the limitations met with dietary questionnaires. It is expected that these biomarkers will be increasingly used in cohort studies to evaluate the effects of non-alcoholic beverages on disease risk. However, it will also be important to carefully evaluate the respective advantages of biomarkers and questionnaires, an evaluation that has not been done yet.

## Additional files

Additional file 1: **Figure S1.** Flow chart of literature search and screening for papers on biomarkers for non-alcoholic beverages. A, coffee; B, tea; C, low-calorie-sweetened beverages. (DOCX 134 kb)

Additional file 2: **Table S1.** Low-calorie sweeteners approved for use in the European Union. (DOCX 16 kb)

## Abbreviations

AUC: Area under the curve
BVO: Brominated vegetable oil
C: Catechin
CG: Catechin gallate
EC: Epicatechin
ECG: Epicatechin gallate
EGC: Epigallocatechin
EGCG: Epigallocatechin gallate
EPIC: European Prospective Investigation into Cancer and Nutrition
FFQ: Food frequency questionnaire
GC: Gallocatechin
GCG: Gallocatechin gallate
GC-MS: Gas chromatography – mass spectrometry
HFCS: High-fructose corn syrup
LC-MS: Liquid chromatography – mass spectrometry
LCS: Low-calorie sweeteners
LCSB: Low-calorie-sweetened beverages
NHDC: Neohesperidine dihydrochalcone
NMR: Nuclear magnetic resonance
ROC: Receiver operating characteristic
SSB: Sugar-sweetened beverages
