# Supplementary material for: Biomarkers of intake for coffee, tea, and sweetened beverages
Source: Genes Nutr. 2018 Jul 4;13:15. doi: 10.1186/s12263-018-0607-5 (PMC6030755; doi:10.1186/s12263-018-0607-5)

**Figure S1**. Flow chart of literature search and screening for papers on biomakers for non-alcoholic beverages. A, Coffee; B, Tea; C, Low-calorie sweetened beverages.

A


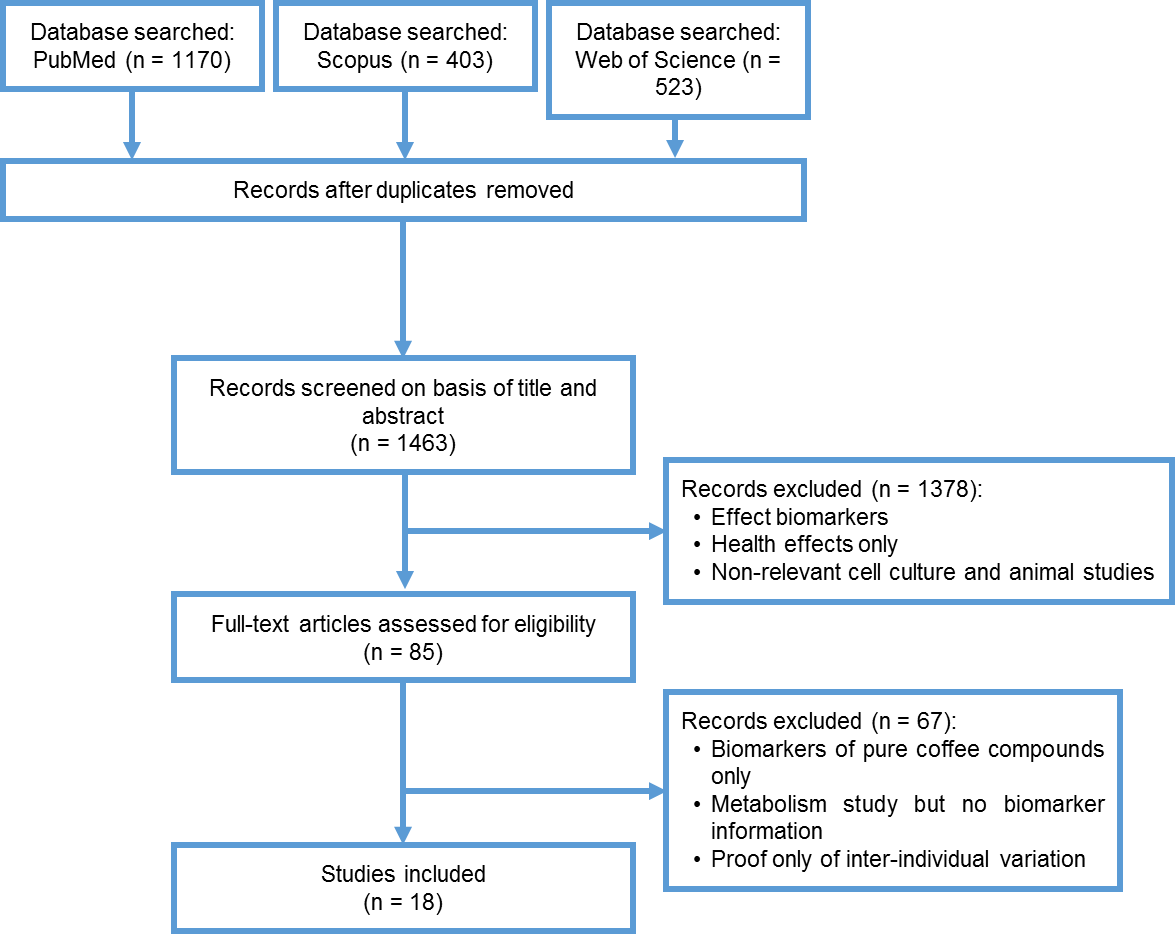


**Figure S1**. Continued.

B


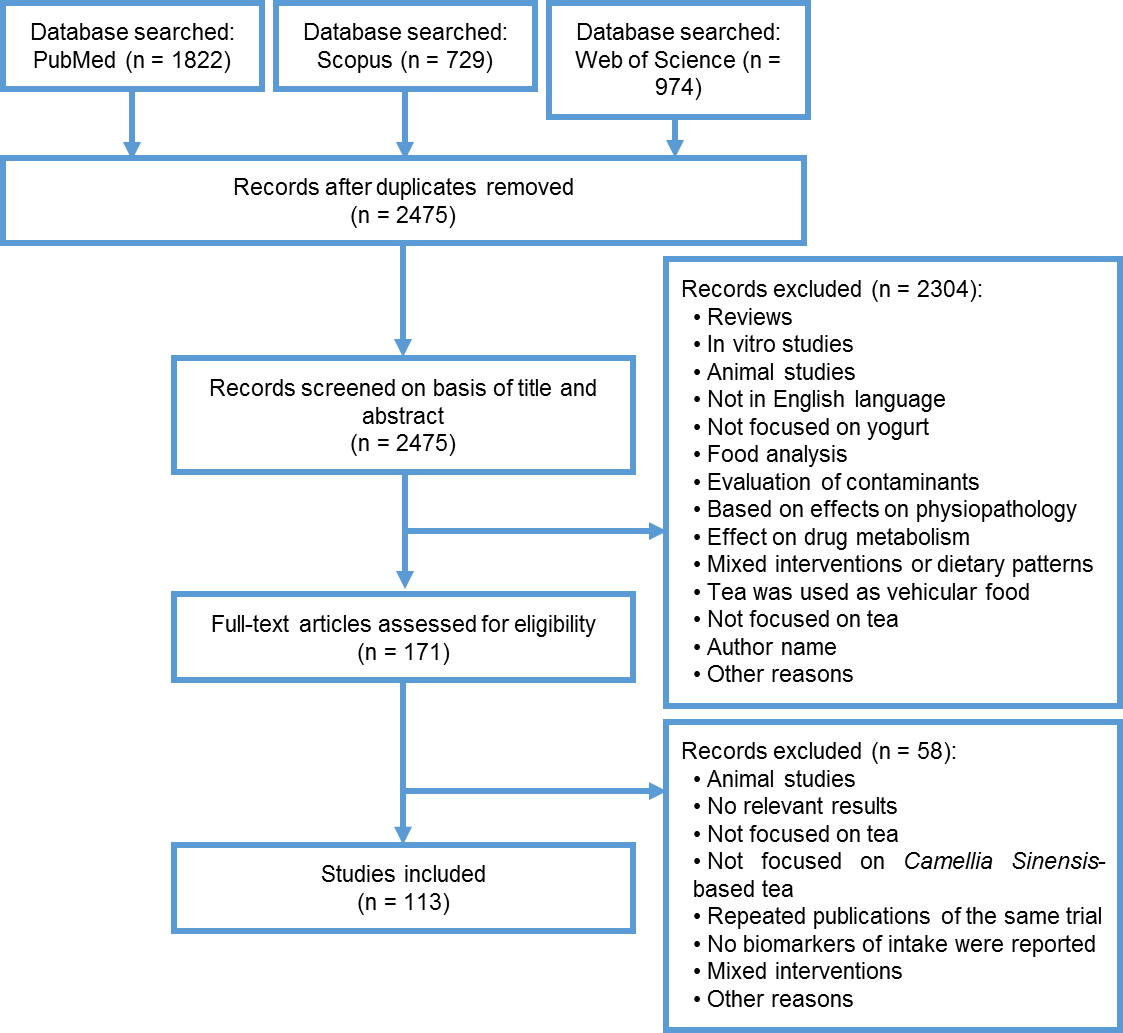


**Figure S1**. Continued.

C


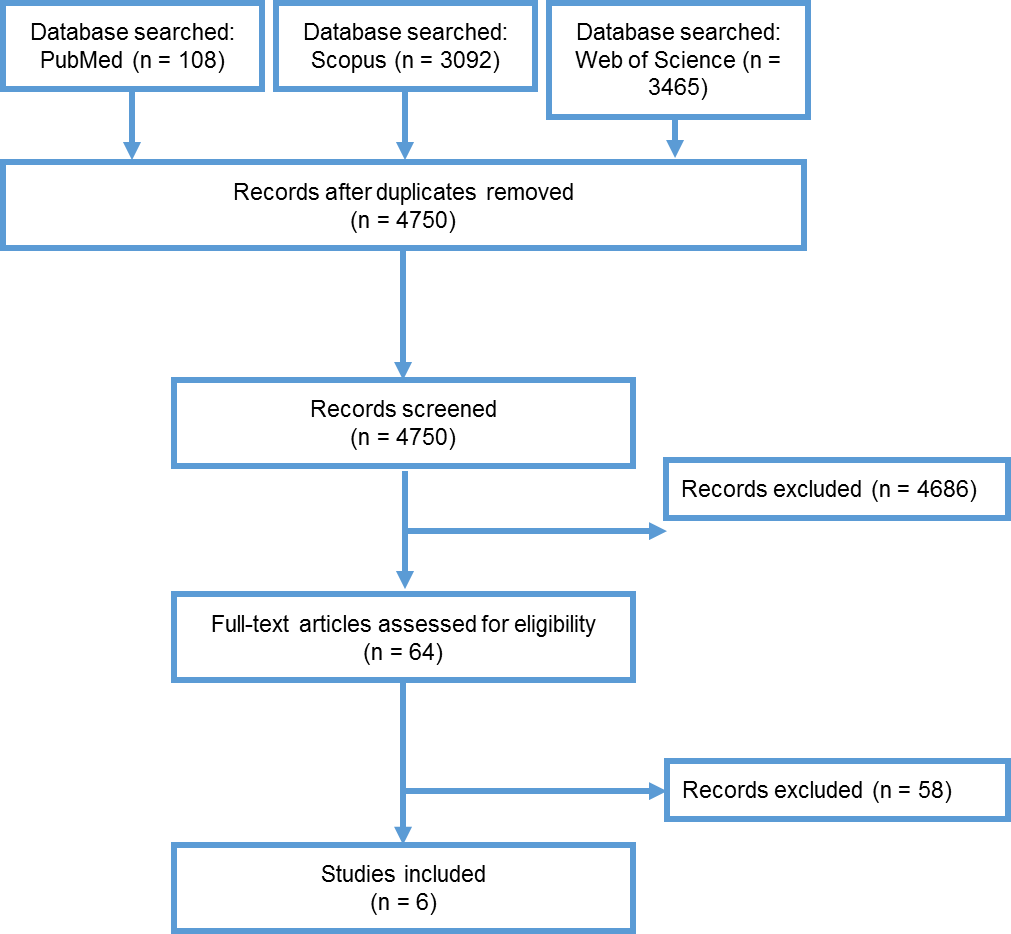

Supplement: Supplementary file 1 — Figure S1. Flow chart of literature search and screening for papers on biomarkers for non-alcoholic beverages. A, coffee; B, tea; C, low-calorie-sweetened beverages. (DOCX 134 kb) [file 12263_2018_607_MOESM1_ESM.docx]
