# Supplementary material for: Biomarkers of intake for coffee, tea, and sweetened beverages
Source: Genes Nutr. 2018 Jul 4;13:15. doi: 10.1186/s12263-018-0607-5 (PMC6030755; doi:10.1186/s12263-018-0607-5)
Supplement: Supplementary file 2 — Table S1. Low-calorie sweeteners approved for use in the European Union. (DOCX 16 kb) [file 12263_2018_607_MOESM2_ESM.docx]

**Table S1**. Low-calorie sweeteners approved for use in the European Union.

| **Intense sweeteners** | **E-number** | **Sweetness^*^** | **ADI (mg/kg BW)** | **Year of approval** |
| --- | --- | --- | --- | --- |
| Saccharin & its salts | E954 | 300-500 | 0-5 | 1977 |
| Aspartame | E951 | 180-200 | 0-40 | 1984 |
| Acesulfame-K | E950 | 200 | 0-9 | 1984 |
| Cyclamates | E952 | 30 | 0-7 | 1984 |
| Thaumatin | E957 | 2000-3000 | No ADI | 1984 |
| NHDC | E959 | 1900 | 0-5 | 1988 |
| Aspartame-acesulfame salt | E962 | 350 | See aspartame & acesulfame-K | 2000 |
| Sucralose | E955 | 600 | 0-15 | 2000 |
| Neotame | E961 | 8000 | 0-2 | 2009 |
| Steviol glycosides | E960 | 300 | 0-4 | 2011 |
| Advantame | E969 | 37000 | 0-5 | 2014 |
| NHDC, neohesperidine dihydrochalcone; ADI, acceptable daily intake; BW, body weight.  ^*^ Relative to sucrose. | | | | |
